# Post-prandial acyl ghrelin infusion in heart failure patients increases gastric emptying rate

**DOI:** 10.1007/s00210-026-05245-5

**Published:** 2026-03-26

**Authors:** Dominic-Luc Webb, Camilla Hage, Ulrika Ljung-Faxén, Gianluigi Pironti, Tonje Thorvaldsen, Daniel C. Andersson, Marcus Ståhlberg, Lars H. Lund, Per M. Hellström

**Affiliations:** 1 Department of Medical Sciences, Gastroenterology and Hepatology, Rudbeck Lab, Uppsala University, Building R3, Floor 4 (Ground Floor), Dag Hammarskjölds Väg 20, 751 85 Uppsala, Sweden; 2 DLW Bioanalytics®, Märsta, Sweden; 3 Department of Medicine, Unit of Cardiology, Karolinska Institutet, Stockholm, Sweden; 4 Heart and Vascular Center, Karolinska University Hospital, Stockholm, Sweden; 5 Perioperative Medicine and Intensive Care, Karolinska University Hospital, Stockholm, Sweden; 6 Department of Physiology and Pharmacology, Karolinska Institutet, Stockholm, Sweden

**Keywords:** Ghrelin, Heart failure, Gastroparesis, Gastric emptying, Acetaminophen, Gastrointestinal agents

## Abstract

Acyl ghrelin increases cardiac output (CO) and contractility in patients with heart failure with reduced ejection fraction (HFrEF). In healthy humans, acyl ghrelin increases gastric emptying rate (GER) and hunger, a potential add-on benefit for HFrEF patients suffering from cachexia with symptoms of delayed gastric emptying and loss of appetite. The aim of this study was to determine if post-prandial acyl ghrelin infusion increases GER and hunger in HFrEF patients compared to CO. HFrEF patients (*n* = 29) arrived fasted and received a 500-kcal breakfast followed by 1.5 g paracetamol. They next received placebo (vehicle, *n* = 15) or acyl ghrelin infusion (0.1 µg/kg/min, *n* = 14) for 120 min. Blood was tapped for plasma at 0, 30, 60, 120, and 150 min into the meal. Hunger scores and CO were recorded. Plasma paracetamol was measured to assess GER. Paracetamol concentration peaked at 30 min in 8 of 14 (57%) in the treatment group versus 1 of 15 (7%) in the placebo group (Mann–Whitney test, *P* = 0.004). Remaining patients peaked at later time points. The acyl ghrelin treatment group data was segregated into rapid (peak at 30 min, *n* = 8) and slow (peak > 30 min, *n* = 6) GER sub-groups. The rapid sub-group had an acyl ghrelin treatment effect on CO (one-way repeat measures, *P* < 0.001) and the highest median increase in hunger across all time points. Acyl ghrelin increases GER and in tandem with increasing CO. Effects on hunger are more difficult to resolve due to variability in subjective scores. Some HFrEF patients may benefit from this add-on effect.

**Supplementary Information:**

The online version contains supplementary material available at 10.1007/s00210-026-05245-5.

## Introduction

Acyl ghrelin (specifically the form octanoylated at Ser-3) is best known as a hunger hormone that decreases in blood concentration following meal ingestion (Kojima and Kangawa 2005; Tschöp et al. 2001). The only known acyl ghrelin receptor is growth hormone secretagogue receptor (GHSR). Acyl ghrelin has pleiotropic effects, accounted for by the broad tissue distribution of GHSR (Akalu et al. 2020). Rodent studies consistently found evidence for gastrointestinal prokinetic effects of acyl ghrelin, including increased gastric emptying rate (GER) of a liquid meal (Dornonville de la Cour et al. 2004; Trudel et al. 2002; Masuda et al. 2000). We demonstrated that post-prandial infusion of acyl ghrelin increased GER of a solid meal containing radiolabeled albumin measured by scintigraphy (i.e., gastric retention was decreased) in normal-weight humans (Levin et al. 2006). These findings were successfully exploited in a human clinical trial seeking to use acyl ghrelin to accelerate GER and time to first bowel movement following open colorectal surgery (Falkén et al. 2013); gastric emptying was determined using paracetamol together with a liquid meal during acyl ghrelin infusion.

Most recently, we demonstrated that infused acyl ghrelin improves cardiac output (CO) in heart failure with reduced ejection fraction (HFrEF) (Erhardsson et al. 2024; Hage et al. 2023; Lund et al. 2023). Gastrointestinal motility impairments, such as gastroparesis, can co-exist with HFrEF, but comorbidity and concomitant treatment of symptoms such as cachexia have not been extensively studied (Yunina et al. 2018). We hypothesized that acyl ghrelin treatment for HFrEF also accelerates GER, resulting in a beneficial add-on effect for HFrEF patients suffering from symptoms such as cachexia and wasting syndrome. In healthy humans, during the first hour of a meal, circulating acyl ghrelin decreases and incretins increase, leading to a transient reduction in GER.

The aim of this study was to determine if infused acyl ghrelin administered to HFrEF patients during a meal accelerates GER, thus overcoming endogenous signals (e.g., incretins) that normally delay GER. This would presumably be when infused acyl ghrelin would have the greatest obstacle to increasing GER, thus representing a rigorous test of acyl ghrelin to increase GER.

## Methods

Acyl ghrelin for infusion was product U-1250, 4071,265 from Clinalfa® product line (Bachem, Bubendorf, Switzerland). This is octanoylated at Ser-3 (CAS No: 258279–04–8). This compound is the same as “ghrelin” used elsewhere according to guidelines of the Ghrelin Nomenclature Consensus Group (Perelló et al. 2023).

### Study subjects

HFrEF patients derive from the Karolinska Acyl Ghrelin Trial (ClinicalTrials.gov NCT05277415). This was a double-blind placebo-controlled trial of acyl ghrelin to treat HFrEF. Subjects and interventions herein are the same as earlier publications where they have been described in detail (Erhardsson et al. 2024; Hage et al. 2023; Lund et al. 2023). Exclusion criteria are in Lund et al. (2023, Supplement 1), among which were point 12, current hormonal treatment; point 13, current immunosuppressive treatment other than corticosteroids; and point 14, any inotropes within 2 weeks prior to study day. Current co-medication data is in Table 1 of that same publication. Two of the originally recruited 31 patients were excluded because they did not take the paracetamol, leaving 29 patients investigated for GER.

**Table 1:** Baseline characteristics. Data is median (IQR)

Variable	Ghrelin Infusion		Placebo *n* = 15
	Rapid GER *n* = 8	Slow GER *n* = 6	
Age	71 (66–74)	65 (59–73)	72 (56–75)
BMI	29 (27–31)	27 (26–29)	30 (28–32)
LVEF	30 (23–36)	27 (23–27)	24 (16–30)
SBP	104 (85–115)	99 (95–105)	105 (92–127)
HR	70 (59–73)	74 (69–85)	63 (59–82)
NT-proBNP	2510 (863–7895)	2190 (1790–3210)	2180 (780–4630)
eGFR	62 (54–78)	61 (46–66)	62 (47–71)

### Ethics

This study was approved by the local (Stockholm) ethics committee (2008/1:12 and 2008/1695–31). Because this was a study of an endogenous peptide, the committee waived the need for medical products agency (MPA) approval, based on a previous waiver for the same treatment in another study of gastrointestinal effects of acyl ghrelin (MPA waiver number 159:2007/16373; ethics Stockholm number 2007/119–31/1). All patients provided written informed consent.

### Gastric emptying (paracetamol absorption test)

Patients arrived fasted in the morning and received a 500-kcal mixed composition breakfast. Following this, in the minute preceding start of randomized intravenous treatments, patients received 1.5 g paracetamol (3 × 500 mg tablets, Alvedon®, GlaxoSmith Kline, Middlesex Brentford, UK). Patients next received placebo (vehicle, *n* = 15) or acyl ghrelin infusion (0.1 µg/kg/min, *n* = 14) for 120 min. Blood was collected for plasma at 0 (baseline), 30, 60, 120, and 150 min following the meal as well as at follow-up 2–5 days later. The clinical chemistry unit paracetamol assay was initially used, which had a lower limit of quantification (LLOQ) of 20 µM. Two patients in the acyl ghrelin group had several samples at or below this cutoff. Therefore, samples were re-run using LC–MS (LLOQ ~ 40 nM). For LC–MS, 25 µl plasma was extracted by addition of 400 µl 20% methanol, liberating bound paracetamol. This was centrifuged at 2500 RCF, 4 °C, 10 min. Supernatant was run through solid phase extraction (Hypersep C8, Cat 60,108–392, Thermo Fisher Scientific, Uppsala, Sweden), eluting with 20% methanol. Void volume containing paracetamol was dried in a speedvac and reconstituted in 100 µl H_2_O. This went to LC–MS (single ion recording, positive mode, 152.0 m/z, representing the M + H (i.e., M + 1) species). The LC–MS rig was Waters Acquity UPLC H-class with QDA detector and Empower 3 software. Concentrations were determined using AUC of µV/s peaks of pure paracetamol standards (Sigma-Aldrich, Cat. A5000, St Louis, MO, USA). The protocol was validated with spiked plasma controls. This assay reflects total (i.e., free + bound) blood concentration.

### Cardiac output

Non-invasive resting CO was assessed in duplicate at time points 0, 60, and 120 min after start and 30 min after stop of infusion (150 min elapsed time) using the Innocor® device (Innovision, Odense, Denmark). Innocor® measures pulmonary blood flow by inert gas rebreathing technique corrected for intrapulmonary shunt, which has been shown to yield a reliable estimate of CO (Ståhlberg et al. 2009).

### Appetite and hunger scores

Before infusion, appetite was marked on a 0–100-mm scale with the following questions: (a) Hunger: “not hungry at all” and “maximal hunger”; (b) Fullness: “not full at all” and “full”; (c) Desire to eat: “no desire to eat” and “extreme desire to eat”; (d) Prospective consumption: “nothing” and “as much as possible.” Hunger scores were collected at the same time points as blood taps for paracetamol. This was a 0- to 10-point visual analog scale. Data was normalized to baseline (0 min).

### Statistics

Total amount of paracetamol absorption was calculated from µM concentration versus time (min) as AUC using the linear trapezoid method. Two-way ANOVA with repeat measures was pursued for un-stratified paracetamol time series and hunger data. Because datasets can fail both normality and equal variance tests, additional statistical tests were also done (e.g., Mann–Whitney). GER was also quantified as time to peak. Pass/fail-type comparisons (e.g., peak reached versus not reached by 30 min) were done by the Mann–Whitney rank-sums test. Changes in CO relative baseline in rapid and slow GER were by one-way repeat measures.

## Results

### Post-prandial acyl ghrelin infusion increases gastric emptying

**Fig. 1:**
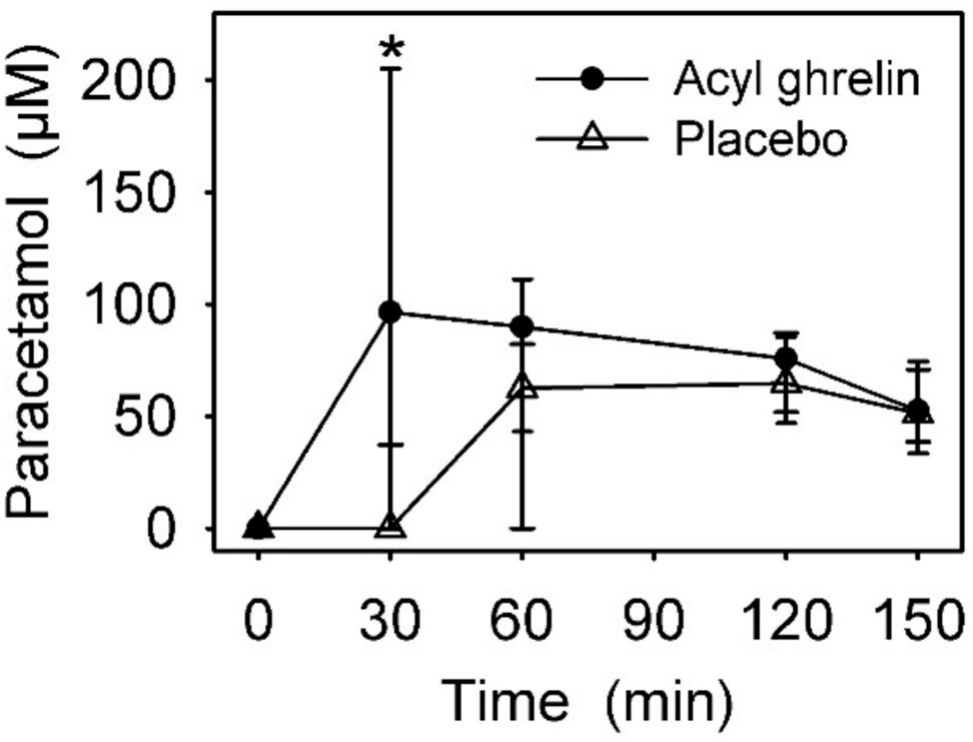
Ghrelin infusion results in higher paracetamol concentration early in a meal. Data is median and IQR. An asterisk indicates *P* < 0.05

The time course of paracetamol concentrations in the HFrEF patients is shown in Fig. 1. Skewness in paracetamol AUC_0–150 min_ data was trivial; mean values differed from median by only 7% (placebo) and 4% (acyl ghrelin treatment) groups (Supplement 1, Sect. 1). Median AUC_0–150 min_ was higher in the acyl ghrelin treatment group, but barely reached statistical significance (Mann–Whitney, *P* = 0.038). There was also considerable overlap in AUC_0–150 min_. Of the acyl ghrelin group, three (21%) had AUC_0–150 min_ below median of placebo group, whereas four (27%) subjects in the placebo group had AUC_0–150 min_ above median of the acyl ghrelin group. This implies that total paracetamol absorption up to 150 min was similar between the groups and representative for gastric emptying.

**Fig. 2:**
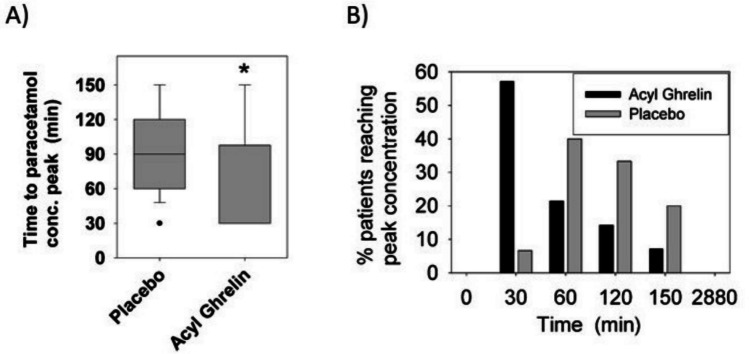
Time to peak and distribution of peak paracetamol concentration in HFrEF patients indicates acyl ghrelin increased GER. **A** Time to peak paracetamol concentration was lower in the acyl ghrelin infusion group (30, 30–90 min) (median, IQR) than the placebo group (90, 60–120 min), *P* < 0.05. Box plot is median and 10, 25, 75, and 90 percentile; dot is outlier. **B** A higher percentage of the acyl ghrelin infusion group than placebo reached peak paracetamol concentration by 30 min. The placebo group lagged behind the acyl ghrelin treatment group at all subsequent times. There were no anomalous peaks at 0 min baseline. At follow-up 2–5 days later (labeled “2880” min), paracetamol was not detected in the placebo group and was 0 ± 17 µM in the acyl ghrelin treatment group as a result of detection in four subjects. No false positives were found during validation of paracetamol assays. The *X*-axis is scaled as category to optimally display all time points. Bars are percent of patients within each group that peaked at indicated blood draw time point

In healthy subjects, the highest paracetamol concentration would be expected at 30 min. Paracetamol concentrations at 30 min were higher in the acyl ghrelin group [96.5 (0–204) µM] [median (IQR)] versus placebo [0 (0–35.7)] (Mann–Whitney, *P* = 0.046). Further analysis by two-way ANOVA with repeated measures revealed that key differences between placebo versus acyl ghrelin were within 30 min (difference of means 83.7 µM, *P* < 0.001) and 60 min (difference of means 29.8 µM, *P* = 0.041). Raw data and analysis are in Supplement 1, Sect. 2. This dataset failed both normality and equal variance tests. Another way to analyze GER across two treatment arms is to sort individuals by time point at which paracetamol reaches peak concentration (i.e., time to peak). Figure 2A summarizes differences in paracetamol time to peak, which was lower in the acyl ghrelin treatment group. As depicted in Fig. 2B, paracetamol peaked at 30 min in 8 of 14 (57%) patients in the acyl ghrelin infusion group versus 1 of 15 (7%) in the placebo group (Mann–Whitney, *P* = 0.004). Hence, acyl ghrelin infusion yielded higher median paracetamol concentration as well as higher likelihood to reach peak at 30 min. Both acyl ghrelin infusion and placebo group had individuals with unexpectedly late peaks after 30 min post infusion (150 min elapsed time that was also latest blood sampling); 2 (14%) in acyl ghrelin group and 3 (20%) in placebo group (Mann–Whitney, *P* = 0.714). The low signals at 0 min baseline as well as 2880 min (follow-up 2–5 days later), and lack of detection of false positives, demonstrated the reliability of the paracetamol assay to determine GER.

The acyl ghrelin infusion group was split into two sub-groups: those who achieved peak paracetamol concentration at 30 min (rapid GER, *n* = 8) and those that peaked at a later time (slow GER, *n* = 6). AUC_0–150 min_ of the rapid GER sub-group [14,555 (84,045–16,153)] [median (IQR)] did not reach significance compared to slow GER sub-group [6935 (4146–11,073)], but it did reach significance compared to placebo [6108 (4938–9837)] (*P* = 0.015). History of diabetes, a recognized contributor to gastroparesis, was distributed similarly across placebo (six cases), slow GER (three cases), and rapid GER (three cases). Two individuals had a history of cachexia, both in the slow GER sub-group. Prior to infusion, a 0–100 VAS score was collected for each of four appetite questions that were summed (SNAQ, max 400), with the following result: placebo 189 (133–207), slow GER 163 (140–232), rapid GER 161 (119–200). Appetite scores were not significantly different.

### Acyl ghrelin increases CO in patients that respond with rapid GER

**Fig. 3:**
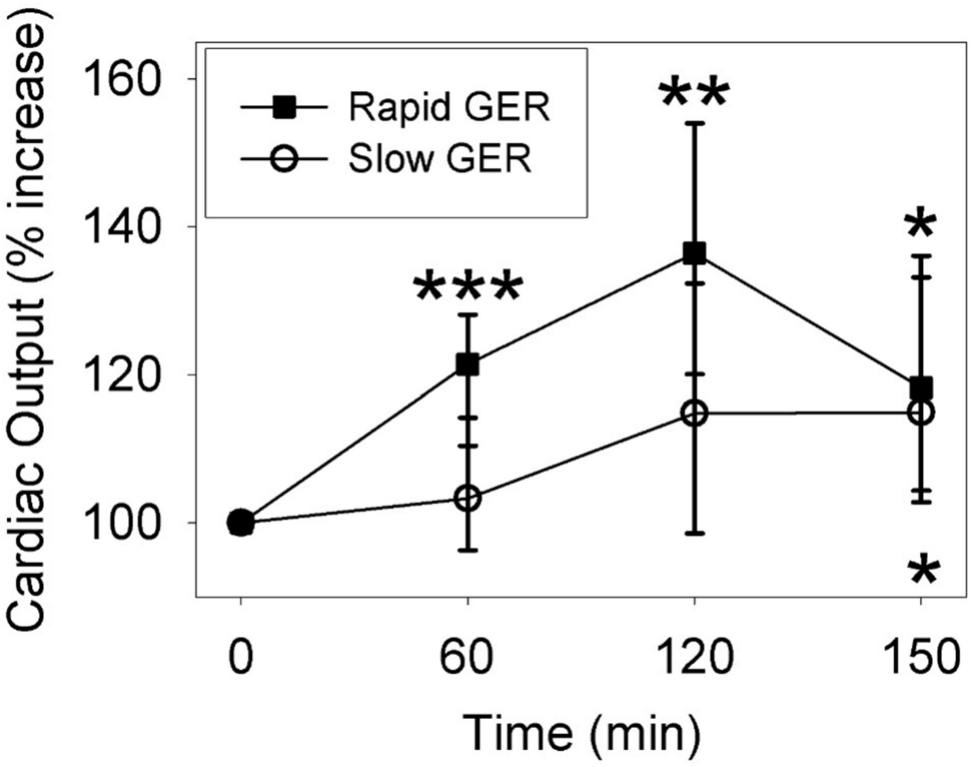
Time course of acyl ghrelin treatment effect on CO in HFrEF patients stratified by rapid or slow GER. Baseline (*t* = 0) CO for the rapid GER sub-group was 4.08 (3.88–4.88) l/min [median (IQR)] and 3.95 (3.35–4.11) l/min for the slow GER sub-group (*P* = 0.31). None of the time points in the slow GER sub-group reached significance compared to baseline. Data points are median and IQR, each patient normalized to own baseline defined as 100%. An asterisk indicates *P* < 0.05, double asterisks indicate *P* < 0.01, triple asterisks indicate *P* < 0.001 in pairwise comparison to baseline within group

Of the entire 29 HFrEF patient cohort, the acyl ghrelin intervention subset with slow GER (paracetamol peak later than 30 min) was most reminiscent of drug-resistant gastroparesis. By one-way repeated measures test, there was an acyl ghrelin treatment effect of increased CO only in the rapid GER sub-group (*P* < 0.001); CO was higher than baseline at 60 (*P* = 0.001), 120 (*P* = 0.004), and 150 (*P* = 0.026) min (Fig. 3). The slow GER sub-group did not have a statistically significant acyl ghrelin treatment effect on CO. In an effort to explain sub-group differences, a selection of baseline characteristics were stratified by rapid and slow GER sub-groups for the acyl ghrelin treatment group (Table 1). There were no clear differences between these sub-groups or placebo.

### Hunger increases over time

**Fig. 4:**
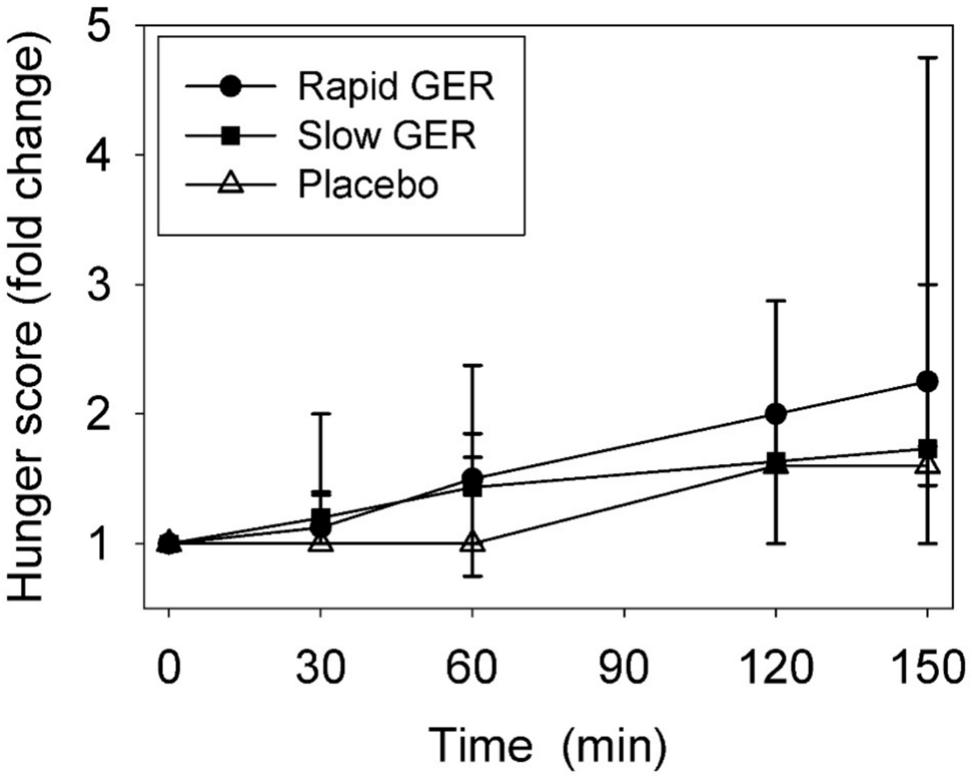
Hunger scores in HFrEF patients treated with acyl ghrelin. Data is stratified by rapid or slow GER and is normalized to baseline (median and IQR). Although the rapid GER group achieved the highest median score by the last time point, there were no significant differences

Fold increase in hunger was highest in the rapid GER subset of the acyl ghrelin–treated group (Fig. 4). However, two-way ANOVA with repeated measures on the normalized dataset did not identify any significant differences. Despite normalization, which tends to reduce variance between groups, the dataset failed equal variance test in addition to normality. To further explore the hunger data, a linear fit for each individual normalized dataset was calculated. Regression coefficients were then used for the Mann–Whitney rank-sum test, with the following results: rapid GER versus slow GER, *P* = 0.18; rapid GER versus placebo, *P* = 0.14; slow GER vs placebo, *P* = 0.91. The coefficient of variation (CV%) between individuals within a group or sub-group often exceeded 50%.

## Discussion

In healthy, fasted humans, infused acyl ghrelin rapidly transduces an effect. Already at 15 min, GH exceeds half maximum concentration, and at 30 min, peak is reached (Broglio et al. 2001). All of the HFrEF patients in the acyl ghrelin infusion group in the present study had a sustained GH response, as it was elevated above baseline at 60 min (Hage et al. 2023). Infused acyl ghrelin appears to have been able to signal through GSHR in all patients in this study.

The experimental setting offered a stringent test of acyl ghrelin to increase GER. One interpretation of the inability of some individuals to achieve rapid GER in response to acyl ghrelin infusion could be that this treatment was simply unable to overcome meal induced incretin effects concomitant with decreased endogenous acyl ghrelin that normally delay GER during this same time window. It is unknown if, or how, this could further explain the comparatively weaker CO responses. Median age and BMI were similar between acyl ghrelin infusion and placebo groups with limited variation (Lund et al. 2023), and there were no statistical differences between rapid and slow GER acyl ghrelin infusion sub-groups or either of these sub-groups against placebo. Metabolic differences were also considered. For example, post-prandial glucose excursions were similar; there were no statistical differences in glucose concentrations at 60 min. In total, over 1200 values were obtained from many different measurements and assays for each patient. None of the current co-medications aligned with the GER findings herein. Nothing further within this collection of data could be identified that might account for the rapid versus slow GER responses to acyl ghrelin infusion. The present findings allow for the possibility that GER and CO are mechanistically linked.

A number of drugs inadvertently inhibit esterases that can deacylate ghrelin, reducing canonical GHS-R1a signaling. The amount of esterase activity in circulation combined with the high concentration of esterase inhibitors needed to protect ghrelin in plasma/serum samples and the non-enzymatic nature of these small molecule inhibitors make it doubtful that any current drug at therapeutic dose can maintain ghrelin concentration. An orally active ghrelin analog seems to be the most straightforward way to enter clinical practice.

In general, cachexia is a recognized and common problem in cardiac patients (Vest et al. 2019), albeit gastroparesis has not been heavily investigated. Prevalence of cachexia has been estimated at 10% (ambulatory cases) to 50% (advanced cases) and perhaps even more than 50% in severe cases (Beltrami et al. 2021; Krysztofiak et al. 2020). The findings herein suggest that a substantial number of cardiac patients might benefit from acyl ghrelin intervention through mitigation of cachexia.

In HF with preserved ejection fraction [HFpEF], obesity is common. Treatment with glucagon-like peptide-1 receptor agonists [GLP1-RAs] reduces weight in obese HFpEF patients. This may have benefits against HFpEF (Kosiborod et al. 2023). HFrEF and HFpEF are different syndromes. GLP1-RAs have not been tested in HFrEF. In HFrEF, particularly with more advanced disease, the benefits of increased contractility and cardiac output may have profound benefits, further enhanced by potential anabolic effects, to improve muscle mass and function (Jankowska and Ponikowski 2023). Future studies should explore this potential add-on effect of ghrelin and GER, and the metabolic benefits vs risks this may entail.

## Strengths, limitations, and future perspectives

We believe this to be the first study to use the paracetamol assay to measure GER in any heart failure patient cohort. It was practical to integrate and revealed differences in GER that could realistically indicate delayed gastric emptying or the effectiveness of a prokinetic intervention. The limited time points for blood draws do not allow exact calculation of time to peak or of how much faster (in min) gastric emptying was with acyl ghrelin infusion. However, this sampling successfully detected accelerated GER using a common blood sampling protocol. The high coefficient of variation of the hunger scores combined with small fold changes ranging between 1.5 and 2.0 fold indicates larger numbers of patients would be needed to reach a high power for hunger score analysis. Normality tests of small samplings from large normally distributed datasets tend to fail normality due to low power. Even with sufficient power and sample size, CO is expected to fail normality tests due to true biological variation. Paracetamol datasets with n > 10, especially within the 30–120-min time window where differences between groups are found, are usually normally distributed (e.g., Shapiro–Wilk test). The varied GER responses to infused acyl ghrelin in HFrEF reported herein were unknown prior to this study. The present findings indicate that future studies of stratified GER groups would benefit from larger sample sizes, perhaps a minimum of *n* = 15 for each stratified group.

## Conclusions

Acyl ghrelin infusion accelerates GER in many HFrEF patients during a meal when endogenous acyl ghrelin normally declines and incretins normally rise. Some of these patients could also suffer from delayed gastric emptying and would benefit from the add-on effect of accelerated GER with acyl ghrelin treatment. The detection of slow GER despite acyl ghrelin infusion from a cohort of only 29 HFrEF patients implies that delayed GER may be common in HFrEF patients. These individuals also had comparatively poor CO responses to acyl ghrelin. Factors associated with poor acyl ghrelin intervention response will need to be identified in order to mitigate this issue.

## Supplementary Information

Below is the link to the electronic supplementary material.

Supplementary Material 1 (PDF 564 KB)

## Data Availability

All source data for this work (or generated in this study) are available upon reasonable request.

## Abbreviations

AUC: Area under the curve
CO: Cardiac output
GER: Gastric emptying rate
GHSR: Growth hormone secretagogue receptor
HFrEF: Heart failure with reduced ejection fraction
IQR: Interquartile range
LC-MS: Liquid chromatography–mass spectrometry
LLOQ: Lower limit of quantification
RCF: Relative centrifugal force
