# Supplementary material for: Post-prandial acyl ghrelin infusion in heart failure patients increases gastric emptying rate
Source: Naunyn Schmiedebergs Arch Pharmacol. 2026 Mar 26;399(9):13437–43. doi: 10.1007/s00210-026-05245-5 (PMC13357392; doi:10.1007/s00210-026-05245-5)
Supplement: Supplementary file 1 — Supplementary Material 1 (PDF 564 KB) [file 210_2026_5245_MOESM1_ESM.pdf]

Section 1. Paracetamol concentration versus time AUC in HFrEF patients infused with ghrelin or vehicle. AUC was calculated using the linear trapezoidal rule in Excel as follows: Area = (Conc2+Conc1)/2*(Time2-Time1). Areas calculated from µM paracetamol concentrations at T=0, 30, 60, 120 and 150 min were summed to obtain AUC_{0-150}.

Group	N	Median	IQR
Ghr_AUC_{0-150}	14	10112	6168 - 15865
Plac_AUC_{0-150}	15	6108	4938- 9837

Mann-Whitney U Statistic= 57.000

T = 258.000 n(small)= 14 n(big)= 15 (P = 0.038)

Group	N	Median	IQR
Rapid_AUC_{0-150}	8	14555	8405 - 16153
Plac_AUC_{0-150}	15	6108	4938 - 9837

Mann-Whitney U Statistic= 22.000

T = 134.000 n(small)= 8 n(big)= 15 (P = 0.015)

Group	N	Median	IQR
Slow_AUC_{0-150}	6	6935	4146 - 11073
Plac_AUC_{0-150}	15	6108	4938 - 9837

Mann-Whitney U Statistic= 35.000

T = 76.000 n(small)= 6 n(big)= 15 (P = 0.460)

Group	N	Median	IQR
Rapid_AUC_{0-150}	8	14555	8405 - 16153
Slow_AUC_{0-150}	6	6935	4146 - 11073

Mann-Whitney U Statistic= 9.000

T = 30.000 n(small)= 6 n(big)= 8 P(est.)= 0.061 P(exact)= 0.059

Section 2. Paracetamol concentration analysis by two-way ANOVA with repeat measures.

Input data.

Subject ID	FactorA Treatment	FactorB Time min	Data Parac µM
3001	Ghrelin	-60	0
3001	Ghrelin	0	0
3001	Ghrelin	30	0
3001	Ghrelin	60	153
3001	Ghrelin	120	64
3001	Ghrelin	150	44
3004	Ghrelin	-60	0
3004	Ghrelin	0	0
3004	Ghrelin	30	0
3004	Ghrelin	60	51
3004	Ghrelin	120	76
3004	Ghrelin	150	47
3005	Ghrelin	-60	0
3005	Ghrelin	0	0
3005	Ghrelin	30	104
3005	Ghrelin	60	61
3005	Ghrelin	120	38
3005	Ghrelin	150	24
3007	Ghrelin	-60	0
3007	Ghrelin	0	0
3007	Ghrelin	30	0
3007	Ghrelin	60	1
3007	Ghrelin	120	2
3007	Ghrelin	150	4
3009	Ghrelin	-60	0
3009	Ghrelin	0	0
3009	Ghrelin	30	30
3009	Ghrelin	60	20
3009	Ghrelin	120	8
3009	Ghrelin	150	16
3010	Ghrelin	-60	0
3010	Ghrelin	0	0

3010	Ghrelin	30	121
3010	Ghrelin	60	73
3010	Ghrelin	120	53
3010	Ghrelin	150	37
3014	Ghrelin	-60	0
3014	Ghrelin	0	0
3014	Ghrelin	30	40
3014	Ghrelin	60	77
3014	Ghrelin	120	50
3014	Ghrelin	150	37
3015	Ghrelin	-60	0
3015	Ghrelin	0	0
3015	Ghrelin	30	0
3015	Ghrelin	60	0
3015	Ghrelin	120	86
3015	Ghrelin	150	106
3018	Ghrelin	-60	0
3018	Ghrelin	0	0
3018	Ghrelin	30	256
3018	Ghrelin	60	165
3018	Ghrelin	120	125
3018	Ghrelin	150	100
3020	Ghrelin	-60	20
3020	Ghrelin	0	0
3020	Ghrelin	30	209
3020	Ghrelin	60	112
3020	Ghrelin	120	80
3020	Ghrelin	150	61
3021	Ghrelin	-60	0
3021	Ghrelin	0	0
3021	Ghrelin	30	233
3021	Ghrelin	60	110
3021	Ghrelin	120	76
3021	Ghrelin	150	58
3023	Ghrelin	-60	20
3023	Ghrelin	0	0
3023	Ghrelin	30	89
3023	Ghrelin	60	104
3023	Ghrelin	120	101

3023	Ghrelin	150	82
3026	Ghrelin	-60	0
3026	Ghrelin	0	0
3026	Ghrelin	30	146
3026	Ghrelin	60	103
3026	Ghrelin	120	76
3026	Ghrelin	150	58
3029	Ghrelin	-60	0
3029	Ghrelin	0	0
3029	Ghrelin	30	204
3029	Ghrelin	60	111
3029	Ghrelin	120	85
3029	Ghrelin	150	72
3002	Placebo	-60	0
3002	Placebo	0	0
3002	Placebo	30	0
3002	Placebo	60	0
3002	Placebo	120	91
3002	Placebo	150	56
3003	Placebo	-60	0
3003	Placebo	0	0
3003	Placebo	30	31
3003	Placebo	60	104
3003	Placebo	120	74
3003	Placebo	150	62
3006	Placebo	-60	0
3006	Placebo	0	0
3006	Placebo	30	112
3006	Placebo	60	82
3006	Placebo	120	57
3006	Placebo	150	42
3008	Placebo	-60	0
3008	Placebo	0	0
3008	Placebo	30	0
3008	Placebo	60	0
3008	Placebo	120	91
3008	Placebo	150	75
3011	Placebo	-60	0
3011	Placebo	0	0

3011	Placebo	30	0
3011	Placebo	60	36
3011	Placebo	120	53
3011	Placebo	150	34
3012	Placebo	-60	0
3012	Placebo	0	0
3012	Placebo	30	0
3012	Placebo	60	77
3012	Placebo	120	43
3012	Placebo	150	0
3013	Placebo	-60	0
3013	Placebo	0	0
3013	Placebo	30	0
3013	Placebo	60	63
3013	Placebo	120	61
3013	Placebo	150	39
3016	Placebo	-60	0
3016	Placebo	0	0
3016	Placebo	30	40
3016	Placebo	60	129
3016	Placebo	120	87
3016	Placebo	150	68
3017	Placebo	-60	0
3017	Placebo	0	0
3017	Placebo	30	0
3017	Placebo	60	0
3017	Placebo	120	24
3017	Placebo	150	153
3019	Placebo	-60	0
3019	Placebo	0	0
3019	Placebo	30	0
3019	Placebo	60	0
3019	Placebo	120	0
3019	Placebo	150	47
3022	Placebo	-60	0
3022	Placebo	0	0
3022	Placebo	30	20
3022	Placebo	60	64
3022	Placebo	120	67

3022	Placebo	150	47
3024	Placebo	-60	0
3024	Placebo	0	0
3024	Placebo	30	0
3024	Placebo	60	69
3024	Placebo	120	65
3024	Placebo	150	51
3025	Placebo	-60	0
3025	Placebo	0	0
3025	Placebo	30	0
3025	Placebo	60	0
3025	Placebo	120	80
3025	Placebo	150	107
3030	Placebo	-60	0
3030	Placebo	0	0
3030	Placebo	30	38
3030	Placebo	60	105
3030	Placebo	120	91
3030	Placebo	150	71
3031	Placebo	-60	0
3031	Placebo	0	0
3031	Placebo	30	37
3031	Placebo	60	47
3031	Placebo	120	52
3031	Placebo	150	36

SigmaPlot 11.0 screenshot of settings for two way ANOVA with repeat measures.

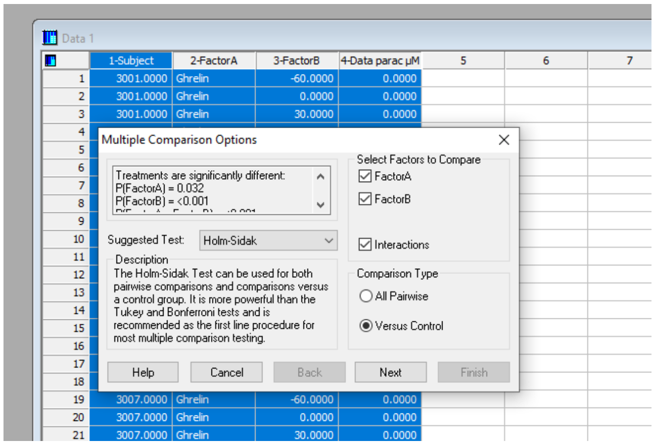

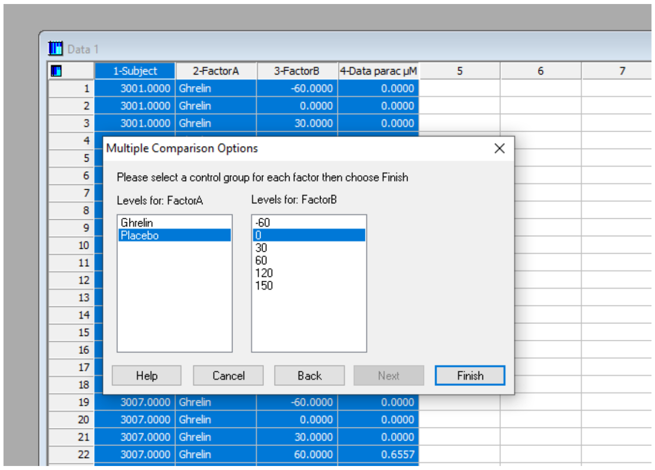

SigmaPlot 11.0 output.

Two Way Repeated Measures ANOVA (One Factor Repetition) fredag, juni 14, 2024, 09:32:40

Data source: Data 1 in 2wayANOVA_w_RM_13jun2024

General Linear Model

Dependent Variable: Data parac µM

Normality Test (Shapiro-Wilk) Failed (P < 0.050)

Equal Variance Test: Failed (P < 0.050)

**Source of Variation**	**DF**	**SS**	**MS**	**F**	**P**
FactorA	1	15607.321	15607.321	5.134	0.032
Subject(FactorA)	27	82075.683	3039.840		
FactorB	5	145814.468	29162.894	24.316	<0.001
FactorA x FactorB	5	41937.217	8387.443	6.993	<0.001
Residual	135	161911.808	1199.347		
Total	173	445357.105	2574.319		

Main effects cannot be properly interpreted if significant interaction is determined. This is because the size of a factor's effect depends upon the level of the other factor.

The effect of different levels of FactorA depends on what level of FactorB is present. There is a statistically significant interaction between FactorA and FactorB. (P = <0.001)

Power of performed test with alpha = 0.0500: for FactorA : 0.491
Power of performed test with alpha = 0.0500: for FactorB : 1.000
Power of performed test with alpha = 0.0500: for FactorA x FactorB : 0.996

Expected Mean Squares:
Approximate DF Residual for FactorA = 27.000

Expected MS(FactorA) = var(res) + 6.000 var(Subject(FactorA)) + var(FactorA)
Expected MS(Subject(FactorA)) = var(res) + 6.000 var(Subject(FactorA))
Expected MS(FactorB) = var(res) + var(FactorB)
Expected MS(FactorA x FactorB) = var(res) + var(FactorA x FactorB)
Expected MS(Residual) = var(res)

Least square means for FactorA :

Group	Mean	SEM
Ghrelin	50.897	6.016
Placebo	31.944	5.812

Least square means for FactorB :

Group	Mean
-60.000	1.418
0.000	1.320E-015
30.000	60.439
60.000	66.494
120.000	64.018
150.000	56.156

Std Err of LS Mean = 7.211

Least square means for FactorA x FactorB :

Group	Mean	SEM
Ghrelin x -60.000	2.836	10.372
Ghrelin x 0.000	1.139E-014	10.372
Ghrelin x 30.000	102.291	10.372
Ghrelin x 60.000	81.375	10.372
Ghrelin x 120.000	65.690	10.372
Ghrelin x 150.000	53.192	10.372
Placebo x -60.000	-1.376E-014	10.020
Placebo x 0.000	-1.009E-014	10.020
Placebo x 30.000	18.587	10.020
Placebo x 60.000	51.613	10.020
Placebo x 120.000	62.347	10.020
Placebo x 150.000	59.120	10.020

Multiple Comparisons versus Control Group (Holm-Sidak method):
Overall significance level = 0.05

Comparisons for factor: **FactorA**

**Comparison**	**Diff of Means**	**t**	**P**	**P<0.050**
Placebo vs. Ghrelin	18.953	2.266	0.032	Yes

Comparisons for factor: **FactorB**

**Comparison**	**Diff of Means**	**t**	**P**	**P<0.050**
0.000 vs. 60.000	66.494	7.307	<0.001	Yes
0.000 vs. 120.000	64.018	7.035	<0.001	Yes
0.000 vs. 30.000	60.439	6.642	<0.001	Yes
0.000 vs. 150.000	56.156	6.171	<0.001	Yes
0.000 vs. -60.000	1.418	0.156	0.876	No

Comparisons for factor: **FactorB within Ghrelin**

**Comparison**	**Diff of Means**	**t**	**P**	**P<0.05**
0.000 vs. 30.000	102.291	7.815	<0.001	Yes
0.000 vs. 60.000	81.375	6.217	<0.001	Yes
0.000 vs. 120.000	65.690	5.019	<0.001	Yes
0.000 vs. 150.000	53.192	4.064	<0.001	Yes
0.000 vs. -60.000	2.836	0.217	0.829	No

Comparisons for factor: **FactorB within Placebo**

**Comparison**	**Diff of Means**	**t**	**P**	**P<0.05**
0.000 vs. 120.000	62.347	4.930	<0.001	Yes
0.000 vs. 150.000	59.120	4.675	<0.001	Yes
0.000 vs. 60.000	51.613	4.082	<0.001	Yes
0.000 vs. 30.000	18.587	1.470	0.267	No
0.000 vs. -60.000	3.669E-015	2.902E-016	1.000	No

Comparisons for factor: **FactorA within -60**

**Comparison**	**Diff of Means**	**t**	**P**	**P<0.05**
Placebo vs. Ghrelin	2.836	0.197	0.844	No

Comparisons for factor: **FactorA within 0**

**Comparison**	**Diff of Means**	**t**	**P**	**P<0.05**
Placebo vs. Ghrelin	2.148E-014	1.490E-015	1.000	No

Comparisons for factor: **FactorA within 30**

**Comparison**	**Diff of Means**	**t**	**P**	**P<0.05**
Placebo vs. Ghrelin	83.705	5.804	<0.001	Yes

Comparisons for factor: **FactorA within 60**

**Comparison**	**Diff of Means**	**t**	**P**	**P<0.05**
Placebo vs. Ghrelin	29.762	2.064	0.041	Yes

Comparisons for factor: **FactorA within 120**

**Comparison**	**Diff of Means**	**t**	**P**	**P<0.05**
Placebo vs. Ghrelin	3.343	0.232	0.817	No

Comparisons for factor: **FactorA within 150**

**Comparison**	**Diff of Means**	**t**	**P**	**P<0.05**
Placebo vs. Ghrelin	5.928	0.411	0.682	No
